# Survival Benefit of Surgical Treatment Added Into Systemic Treatment for Isolated Breast Cancer Liver Metastases: A Systematic Review and Meta-Analysis

**DOI:** 10.3389/fsurg.2021.751906

**Published:** 2021-10-25

**Authors:** Ming-Shuai Sun, Hong-Jin Liu, Yong-Yang Yun, Zheng-Heng Yu, Fan Yang, Yin-Hua Liu, Ling Xu

**Affiliations:** Breast Disease Center, Peking University First Hospital, Beijing, China

**Keywords:** breast cancer, liver metastases, surgical treatment, liver resection, survival, meta-analysis

## Abstract

**Background:** Compared with systemic treatment alone, whether surgical treatment combined with systemic treatment can improve survival outcomes of patients with isolated breast cancer liver metastases (BCLM) is still controversial. This meta-analysis was designed to evaluate the efficacy of surgical treatment for patients with isolated BCLM.

**Methods:** A systematic search of PubMed, Embase, and Cochrane Library up to May 13, 2021 was conducted for relevant studies. The primary outcome was overall survival. The meta-analysis was performed using R software. The quality of the pooled study was assessed using the Newcastle-Ottawa scale. The publication bias was evaluated by funnel plots and Begg's and Egger's tests. Fixed- and random-effects models were applied according to heterogeneity.

**Results:** 9 retrospective studies involving 13 cohorts (7 unmatched cohorts and 6 matched cohorts) were included in this study. The surgical cohorts had better overall survival than the systemic cohorts in the pooled analysis of all the included studies, in the subgroup analysis of liver resection, and in the subset of the matched cohorts.

**Conclusions:** Compared with systemic treatment alone, surgical treatment combined with systemic treatment was proven to be associated with superior survival outcomes, which should be considered in selected patients with isolated BCLM.

## Introduction

Breast cancer is the most common tumor in women, and it has even surpassed lung cancer to become the highest incidence of cancer in 2020, according to the latest World Health Organization (WHO) report (1). Liver metastasis (LM) is one of the most common distal organ metastases of breast cancer, and it is also the leading cause of death, with a median overall survival (OS) of 2–3 years (2–4). The traditional treatment of patients with breast cancer liver metastasis (BCLM) involved the use of systemic therapies with the goal of prolonging life, palliating symptoms and improving the quality of life (3). With the development of accurate diagnostic tools and adjuvant systemic therapies, the metastatic diseases can be identified at an earlier stage which are more responsive to treatment. Over the course of time, the mortality rate of metastatic breast cancer (MBC) is decreasing at 1–2% every year (5). Therefore, the aim of treatment is not only palliating symptoms but also delaying disease progression and extending survival (6, 7).

Although systemic therapy is the cornerstone of treatment for metastatic cancer (3), some clinicians have advocated local treatment for some specific metastatic cancers because of the improved effectiveness and decreased complications of local therapeutic techniques (8–10). Currently, local therapeutic approaches are diverse, such as surgical treatment (e.g. resection and ablation) and non-surgical treatment (e.g. stereotactic radiotherapy, intrahepatic chemotherapy, and embolization). Surgical treatment for colorectal liver metastases (CRLM) is now considered as a standard and curable treatment that has been proven to be beneficial to survival for more than 30 years (11). This is due to the unique biology of colorectal cancer that the liver is the first potential metastasis site through splanchnic circulation. In contrast, BCLM would have traveled through the systemic circulation to reach the liver. It is likely that the breast cancer is disseminated by the time liver metastases are diagnosed. Therefore, whether surgical treatment will improve the prognosis of patients with BCLM remains to be determined.

Currently, researches on this subject have been published, but their conclusions were inconsistent and limited by their retrospective nature, single center design, small cohorts, and selection bias. Although previous systematic reviews supported surgical treatment for selected patients with BCLM because their survival outcomes appeared to be superior to systemic treatment alone (8–10), no meta-analysis of pooled data on this topic has been carried out. Therefore, the aim of this meta-analysis is to compare the survival outcomes of patients with isolated BCLM who receive surgical treatment combined with systemic treatment and those who receive systemic treatment alone based on the current available literature.

## Materials and Methods

A study protocol was established prior to the conduct of the systematic review. The objectives, outcomes of interest, search strategy and criteria of inclusion and exclusion were predefined. Methods of quality appraisal were selected following study selection and were based on the nature of the included studies. This meta-analysis was prepared in accordance with the Preferred Reporting Items for Systemic Reviews and Meta-Analysis (PRISMA) statement (12), and the work was reported in line with the Assessing the Methodological Quality of Systematic Reviews (AMSTAR) guidelines (13). This study was registered on PROSPERO (https://www.crd.york.ac.uk/prospero/) with the registration number CRD42021253838, and also registered on INPLASY (https://inplasy.com/) with the registration number INPLASY202150063.

### Search Strategy

A systematic search of the Cochrane Library, Embase, and PubMed was performed to identify relevant studies published up to May 13, 2021. The search strategy was performed using the following medical subject headings and keywords: (“Breast Neoplasms”[Mesh] OR breast) AND (“Liver”[Mesh] OR liver OR hepatic) AND (“Neoplasm Metastasis”[Mesh] OR metastasis OR metastases OR metastatic) AND (“Hepatectomy”[Mesh] OR “Metastasectomy”[Mesh] OR “Radiofrequency Ablation”[Mesh] OR hepatectomy OR metastasectomy OR resection OR ablation OR ablative OR microwave OR surgery OR surgical), see Supplementary Material for full electronic search strategies. The references of relevant reviews and meta-analysis were also manually searched for potentially relevant studies. All relevant studies that met the inclusion criterion were reviewed for data extraction.

### Study Selection

Two authors (Sun MS and Liu HJ) independently scanned the titles and abstracts from the studies identified in the electronic search. Relevant papers were further identified through perusing full texts. Disagreements were resolved by discussion and consensus between authors. Studies were included in this meta-analysis according to the following inclusion criteria: (I) The survival outcomes between patients with isolated BCLM receiving systemic treatment combined with surgical treatment (surgical cohort) and those receiving systemic treatment alone (systemic cohort) were compared. In this study, surgical treatment referred to liver resection (LR) and radiofrequency ablation (RFA), and the term “isolated BCLM” was used to describe BCLM with no extrahepatic lesions except stable bone metastases. (II) The papers were accepted or published, and the full texts were available in English. (III) The outcome of interest was OS, and hazard ratios (HRs) with corresponding 95% confidence intervals (CIs) or survival curves were provided. OS was calculated from date of LM diagnosis to death. The exclusion criteria included were as follows: (I) Non-comparative studies, duplicate studies, articles presented at meetings, review articles, conference abstracts, guidelines, case reports or series, or letters. (II) Comparisons of prognosis with a sample size of less than 20 in either the surgical cohort or the systemic cohort were excluded because they were not statistically effective.

### Quality Assessment

The Newcastle-Ottawa scale (NOS) was used to evaluate the methodological quality of the studies (14). The scale has three parts: Patient selection, comparability, and outcome. Two reviewers (Sun MS and Liu HJ) appraised the quality of studies independently. Disagreements were solved by consensus. The scale changes from 0 to 9 stars, and studies with a score ≥7 stars could be deemed as high quality. The variables related to LM (e.g. time between breast cancer and LM diagnosis, number of LM, maximum diameter of LM) were selected as the most important controlling factor in “comparability”, because previous studies confirmed them as key survival predictors for patients with isolated BCLM and these characteristics significantly influenced the clinical decision-making of treatment (15–18).

### Data Extraction

Two researchers (Sun MS and Liu HJ) reviewed each article by a structured list and extracted data into a database independently. Disagreements in data extraction were resolved by discussion. Data with the following items were extracted: (I) Characteristics including first author, country, publication year, sample size, age of patients, approach of surgical treatment, study years, study design, study center, size and number of LM, and covariates included in matching. (II) The HRs for OS between the surgical cohort and the systemic cohort. When the study reported the HRs, we extracted them directly. When the study reported the HRs of both univariate analysis and multivariate analysis, the HRs of multivariate analysis were extracted. When the study did not report the HRs, we estimated them from the data extracted from the survival curves, as described by Tierney et al. (19).

### Statistical Analysis

The R software version 3.6.3 (http://www.r-project.org) was adopted to perform the meta-analysis. The I2 statistic was utilized to evaluate the heterogeneity among the studies. The random effects model was adopted if I2 was >50%, otherwise the fixed-effects model was utilized. Potential publication bias was determined by a funnel plot and assessed by the Begg's test and Egger's test. In all cases, *P* value < 0.05 was considered statistically significant.

## Results

### Study Characteristics

**Figure 1:**
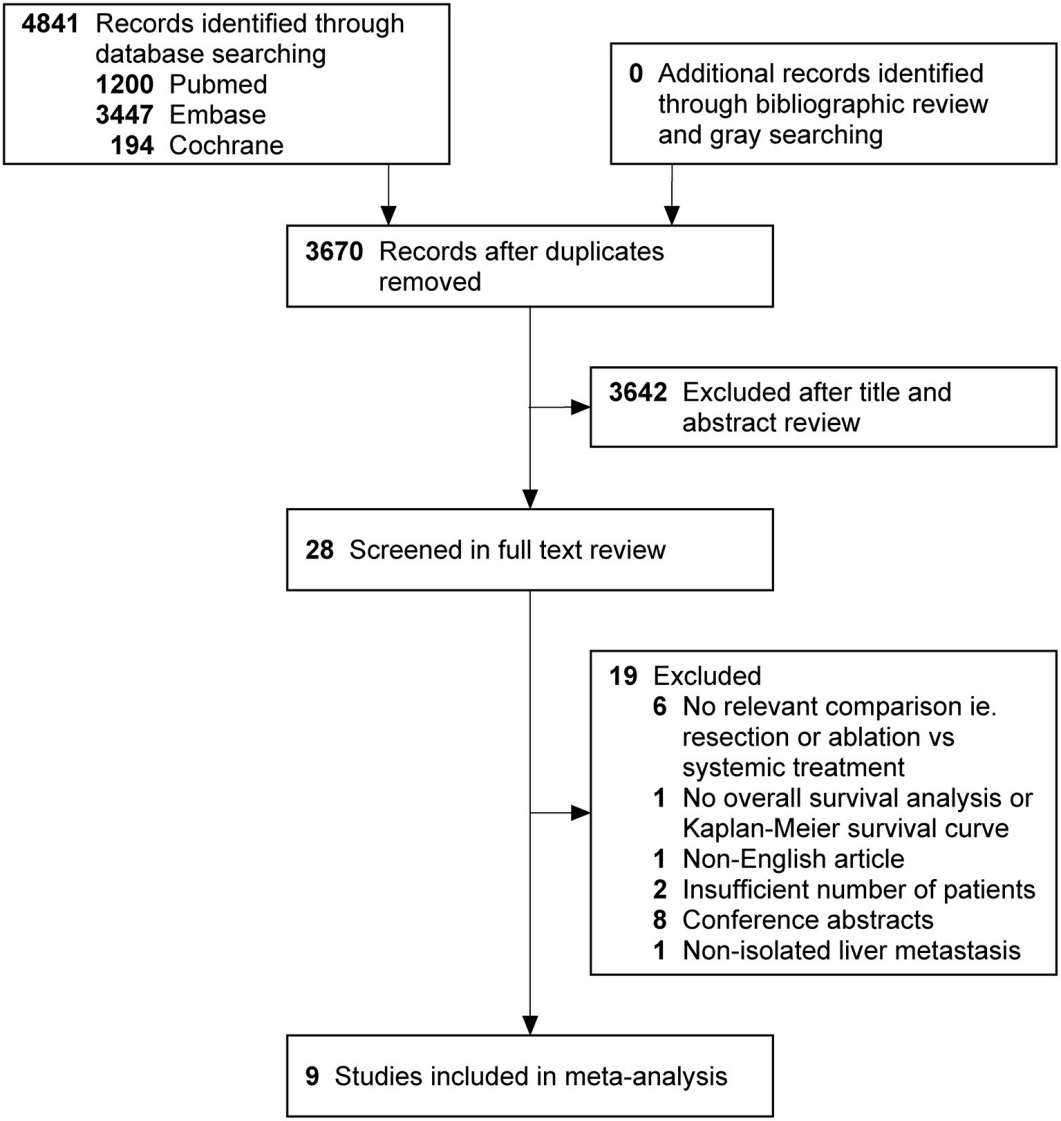
PRISMA flowchart describing literature search history. PRISMA indicates preferred reporting items for systematic reviews and meta-analyses.

The search strategy retrieved 4 841 potentially relevant articles, and no additional records were identified through gray searching. After deduplicating titles and screening abstracts, 28 full-texts were reviewed, of which further 19 studies were excluded for various reasons (Figure 1). Ultimately, nine studies (20–28) were included in this meta-analysis. Characteristics of included studies, matching covariates, and study quality evaluated using the NOS were detailed in Table 1. Surgical treatment was performed by LR in six studies (21–25, 28), by LR or RFA in two studies (20, 26), and by RFA in one (27). The characteristics of patients with isolated BCLM in the study cohorts were summarized in Tables 2, 3. In general, patients who received surgical treatment were younger, more likely to receive breast surgery and have solitary distribution of liver lesions, and had longer intervals from breast cancer diagnosis to BCLM. It needed to be emphasized that Chun et al.'s study (23) included patients with non-osseous extrahepatic metastases in the surgical cohort, which accounted for 9.6%, but no patients in the systemic cohort had any non-osseous extrahepatic metastases. This was due to the vast majority of patients without non-osseous extrahepatic metastases can represent the overall characteristics of the surgical cohort. Besides, considering that our study hypothesis was to prove that systematic treatment combined with surgical treatment can improve the prognoses of patients with isolated BCLM, if the hypothesis could be confirmed in the case of allowing a small number of patients in the surgical cohort with non-osseous extrahepatic metastases, the conclusion will be more credible. Therefore, we allowed this study to be included in this meta-analysis.

**Table 1:** Study demographics, matching variables and quality assessment.

**Study**	**Country**	**Study design**	**Study center**	**Study year**	**Surgical treatment**	**Matching variables^#^**	**Follow-up time (months) (median [range])**	Quality assessment (stars allocated)		
								**Selection**	**Comparability**	**Outcome**
Bilani et al. (24)	America	Retrospective unmatched	Multicenter	2010-2016	LR	–	–	4	0	1
Chun et al. (23)	America	Retrospective matched	Single center	1997-2016	LR	d, f, g, h, i, j, k, l, q, s, t, x	57 (9-229) in LR+ST cohort, 18 (0-213) in ST cohort	4	2	2
Feng et al. (22)	China	Retrospective matched	Single center	2008-2018	LR	Not specific	47.0 (1.5-89.0) in LR+ST cohort, 53.7 (0.1-139.7) in ST cohort	4	2	3
Mariani et al. (28)	France	Retrospective matched	Single center	1988-2007	LR	a, c, e, f, h, i, j	–	4	1	1
Millen et al. (21)	America	Retrospective unmatched	Multicenter	2010-2014	LR	–	–	4	0	1
Ruiz et al. (25)	France and Netherlands	Retrospective matched	Multicenter	1985-2013	LR	b, d, e, r, s, u, v, w	80 in whole cohort, 69 in LR+ST cohort, 80 in ST cohort	3	2	3
Sadot et al. (26)	America	Retrospective matched	Single center	1991-2014	LR/RFA	e, i, m, n, o, p, q	73 in whole cohort, 89 in LR/RFA+ST cohort, 62 in ST cohort	4	2	3
Sunden 2020	Sweden	Retrospective unmatched	Multicenter	2009-2016	LR/RFA	–	–	4	1	2
Tasci 2013	America	Prospective matched	Single center	1996-2011	RFA	e, q, s	20 (6–101) in RFA+ST cohort, 27 in ST cohort	4	2	2

# *Matching variables: Demographics and time interval: a) age at diagnosis of breast cancer, b) age at diagnosis of liver metastases, c) year of breast cancer diagnosis, d) year of liver metastases diagnosis, e) time between breast cancer diagnosis and first liver metastasis. Primary breast tumor: f) TNM stage, g) tumor grade, h) histology, i) ER status, j) PR status, k) HER2 status, l) resection of primary tumor, m) type of breast surgery, n) adjuvant chemotherapy after breast surgery, o) adjuvant radiotherapy after breast surgery, p) targeted therapy. Breast cancer liver metastases: q) number of liver metastases, r) single or multiple metastasis, s) size of liver metastases, t) synchronous liver metastases, u) chemotherapy after diagnosis of liver metastases, v) hormonal therapy after diagnosis of liver metastases, w) targeted therapy after diagnosis of liver metastases, x) best RECIST response to first-line systemic therapy. LR, liver resection; ST, systemic treatment; RFA, radiofrequency ablation*.

**Table 2:** Characteristics of patients with breast cancer liver metastases in the unmatched cohorts (before matching or in studies without matching).

**Study**	**Treatment**	**No. of patients**	**Age at diagnosis (years)^#^**	**Breast surgery (%)**	**Time between BC and LM (months)^#^**	**No. of LM^#^**	**Solitary distribution (%)**	**Maximum size of LM (cm)^#^**	**Non-osseous extrahepatic metastasis (%)**	**Response rate to systemic therapy**	**Median OS (months)** ^†^	**OS rates (1-/3-/5-year) (%)** ^†^
Bilani et al. (24)	LR+ST	101	–	–	–	–	–	–	0%	–	70	–/–/–
	ST	3405	–	–	–	–	–	–	0%	–	26	–/–/–
Chun et al. (23)	LR+ST	136	49 (26-71) for LM	–	19 (0-305)	1 (1-14)	–	2.5 (0.9-14.0)	9.6%	CR+PR=74%	69 (range, 55-83)	–/–/53%
	ST	763	52 (20-92) for LM	–	16 (0-289)	2 (1-10)	–	2.5 (0.8-8.7)	0%	CR+PR=66%	28 (range, 25-31)	–/–/21%
Feng et al. (22)	LR+ST	65	50.9 ± 10.5 for LM	–	50.4 ± 50.8	–	65%	4.1 ± 2.1	0%	CR+PR+SD=100%	–	94.0%/83.2%/58.8%
	ST	319	50.9 ± 10.7 for LM	–	39.4 ± 38.6	–	35%	3.1 ± 2.1	0%	CR+PR+SD=60.4%	32 (95% CI, 28-36)	80.4%/43.0%/28.0%
Millen et al. (21)	LR+ST	83	49.5 ± 11.7	91.6%	–	–	–	–	0%	–	69.7	–/–/–
	ST	1857	54.87 ± 13.0	47.0%	–	–	–	–	0%	–	45.2	–/–/–
Ruiz et al. (25)	LR+ST	139	49.4 ± 10.6 for LM	100%	49.89 ± 42.41	2.32 ± 1.82	40.6%	3.4 ± 1.8	0%	CR+PR+SD=100%	74	–/78%/57%
	ST	523	61.1 ± 14.2 for LM	100%	27.18 ± 15.22	1.98 ± 1.29	13.8%	5.3 ± 2.6	0%	–	13	–/18%/10%
Sadot et al. (26)^‡^	LR/RFA+ST	69 (48 LR, 18 RFA, 3 LR+RFA)	51 (43–59) for LM	100%	53 (27–94)	1 (1–2)	64%	3 (2–5)	0%	–	50	–/–/38%
	ST	98	52 (42–62) for LM	72%	30 (8–46)	3 (1–6)	30%	2.5 (1.4–4.1)	0%	–	45	–/–/39%
Sunden 2020	LR/RFA+ST	29 (24 LR, 5 RFA)	54 (26-78) for LM	–	48 (0-251)	–	65.5%	–	0%	Response rate=90.5%	77 (95% CI, 41-113)	–/–/–
	ST	33	58 (28-86) for LM	–	20 (2-68)	–	69.7%	–	0%	–	28 (95% CI, 13-43)	–/–/–

# *Data are presented as mean ± SD or median (range)*.

† *OS was calculated from date of LM diagnosis to death*.

‡ *Continuous variables in Sadot 2016 are presented as median (interquartile range). BC, breast cancer; LM, liver metastases; BCLM, breast cancer liver metastases; OS, overall survival; LR, liver resection; ST, systemic treatment; RFA, radiofrequency ablation; CI, confidence interval; CR, complete response; PR, partial response; SD, stable disease*.

**Table 3:** Characteristics of patients with breast cancer liver metastases in the matched cohorts.

**Study**	**Treatment**	**No. of patients**	**Age at diagnosis (years)^#^**	**Breast surgery (%)**	**Time between BC and LM (months)^#^**	**No. of LM^#^**	**Solitary distribution (%)**	**Maximum size of LM (cm)^#^**	**Non-osseous extrahepatic metastasis (%)**	**Response rate to systemic therapy**	**Median OS (months)** ^†^	**OS rates (1-/3-/5-year) (%)** ^†^
Chun 2020	LR+ST	72	50 (26-71) for LM	–	–	1 (1-14)	–	2.5 (0.9-14.0)	9.6%	–	–	–/–/56%
	ST	72	51 (25-70) for LM	–	–	2 (1-7)	–	2.5 (0.9-8.5)	0%	–	–	–/–/40%
Feng et al. (22)	LR+ST	33	49.7 ± 12.0 for LM	–	60.0 ± 58.5	–	55%	4.1 ± 2.0	0%	CR+PR+SD=100%	–	92.6%/54.7%/54.7%
	ST	119	51.2 ± 10.9 for LM	–	41.1 ± 41.6	–	50%	3.7 ± 2.6	0%	CR+PR+SD=68.1%	30 (95% CI, 18-43)	79.2%/45.6%/21.9%
Mariani et al. (28)	LR+ST	51	50 (30-69) for BC	–	34 (0-120)	–	–	–	0%	CR+PR+SD=100%	–	–/–/–
	ST	51	51 (29-89) for BC	–	35 (0-216)	–	–	–	0%	CR+PR+SD=100%	–	–/–/–
Ruiz et al. (25)	LR+ST	49	49.8 ± 11.4 for LM	100%	33.45 ± 26.66	2.41 ± 1.63	38.8%	3.2 ± 1.8	0%	CR+PR+SD=100%	82	–/81%/69%
	ST	49	51.9 ± 11.7 for LM	100%	37.18 ± 17.00	2.51 ± 1.54	40.8%	3.8 ± 1.9	0%	–	31	–/33%/24%
Sadot et al. (26)	LR/RFA+ST	49	–	–	–	–	–	–	0%	–	–	–/–/–
	ST	45	–	–	–	–	–	–	0%	–	–	–/–/–
Tasci 2013	RFA+ST	24	50 ± 2, not specific	–	26.5 ± 5.6	2.4 ± 0.4	–	3.7 ± 0.4	0%	–	48	–/–/29%
	ST	32	50 ± 1.8, not specific	–	20.1 ± 3.3	3.3 ± 0.4	–	2.6 ± 0.4	0%	–	9	–/–/0%

# *Data are presented as mean ± SD or median (range)*.

† *OS was calculated from date of LM diagnosis to death. BC, breast cancer; LM, liver metastases; BCLM, breast cancer liver metastases; OS, overall survival; LR, liver resection; ST, systemic treatment; RFA, radiofrequency ablation; CI, confidence interval; CR, complete response; PR, partial response; SD, stable disease*.

### Combined Results of All the Included Studies

**Figure 2:**
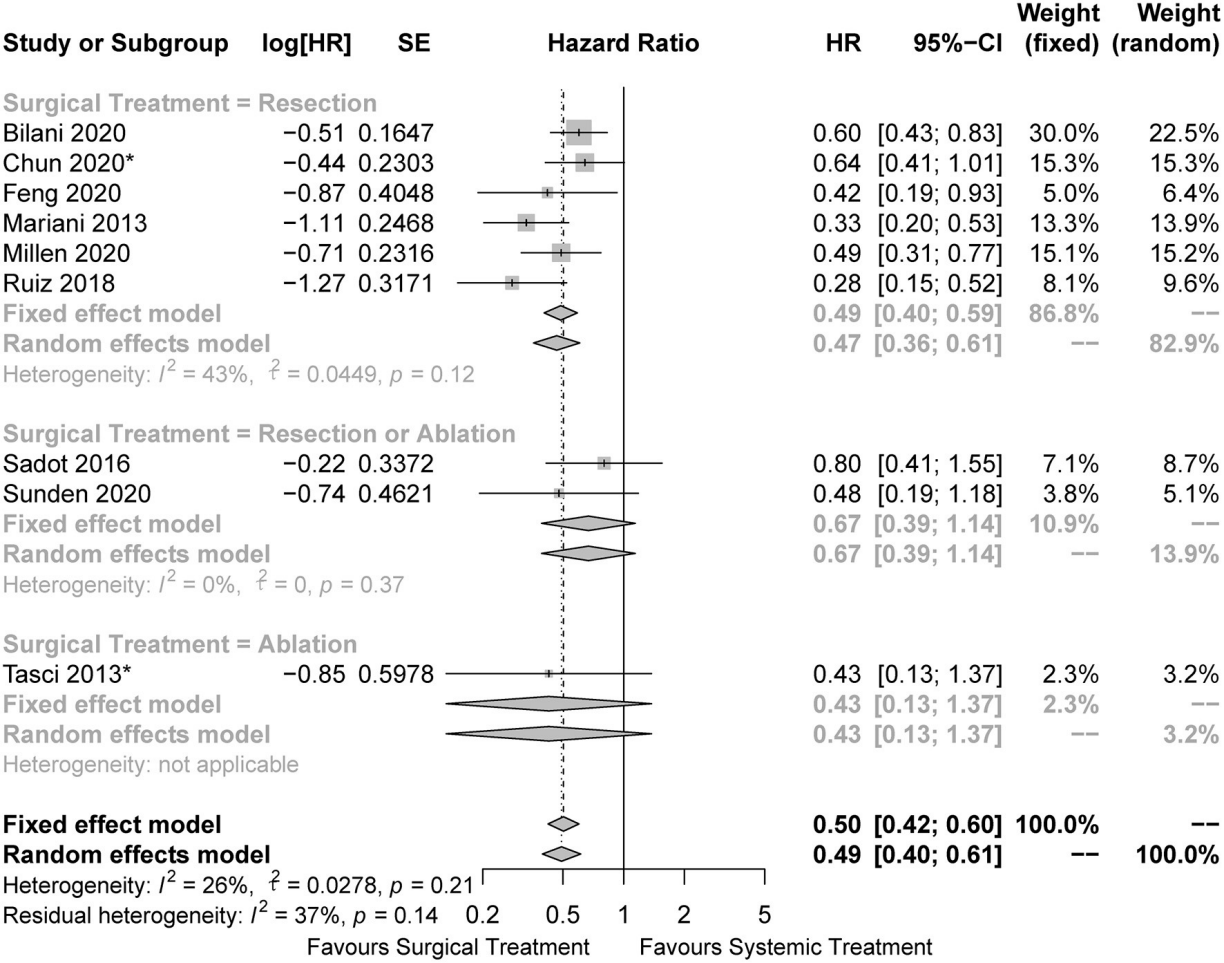
Forest plots for comparison of overall survival between surgical and systemic cohorts in all included studies. The HRs of the matched cohorts were incorporated when the studies also reported the results before matching. *The hazard ratio was estimated from the survival curves.

In the pooled analysis of all the included studies, the surgical cohorts had better OS than the systemic cohorts (HR = 0.50, 95%CI: 0.42 to 0.60, I2 = 26%; Figure 2). In the subgroup analysis, surgical treatment was performed by LR in six studies, of which the pooled analysis also indicated a survival advantage for the LR cohorts (HR = 0.49, 95%CI: 0.40 to 0.59, I2 = 43%; Figure 2). We could not draw conclusions from subsets with other approaches of surgical treatment owing to the limited number of related studies. The results of other survival outcomes (recurrence-free survival, progression-free survival, and disease-free survival) reported in the included studies were summarized in Supplementary Material. Due to the limited number of studies reporting such outcomes and the inconsistent definition of the outcomes, we were unable to perform any pooled analyses regarding these survival outcomes.

### Combined Results of the Matched/Unmatched Cohorts

**Figure 3:**
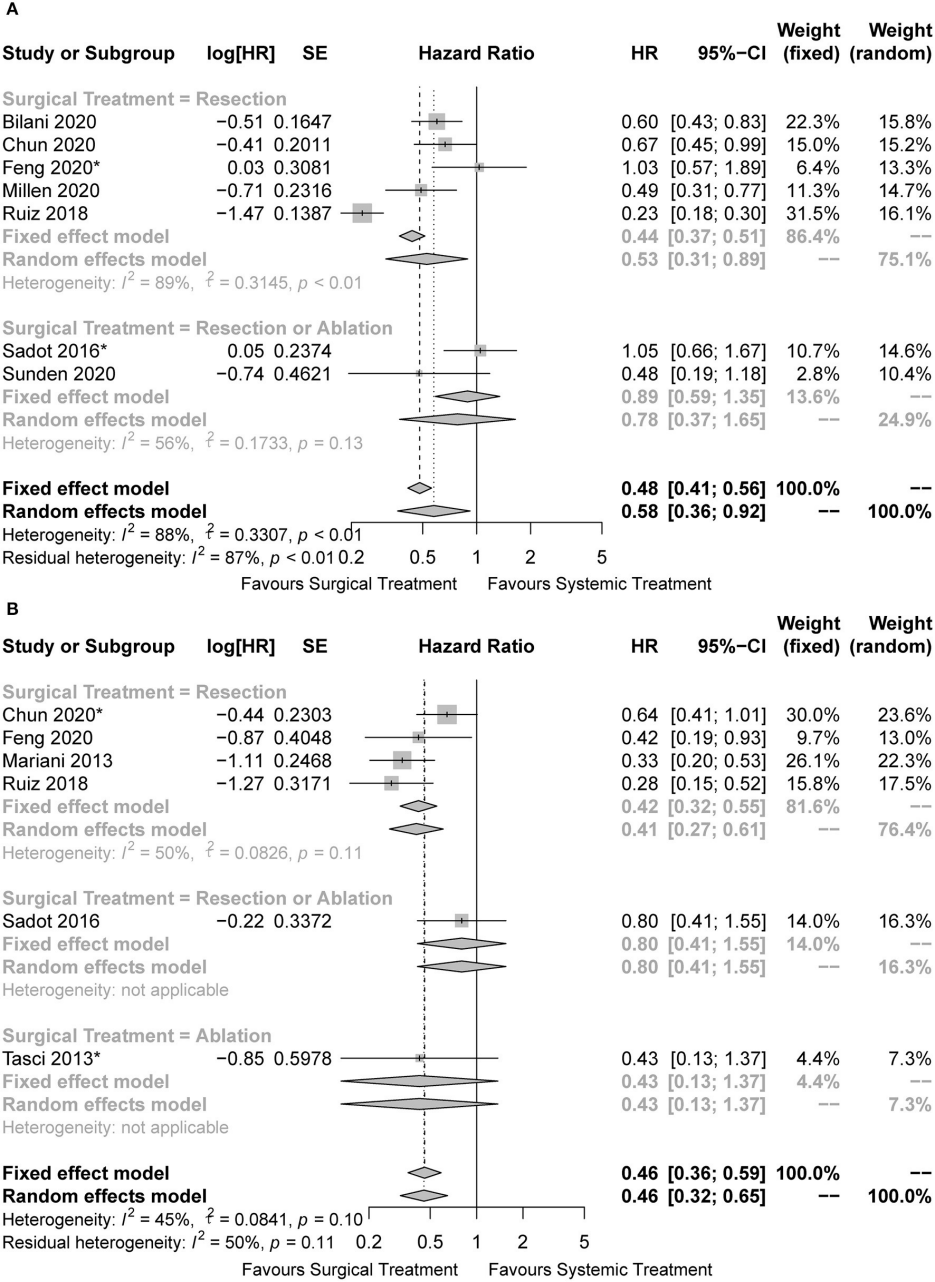
Forest plots for comparison of overall survival between surgical and systemic cohorts in **(A)** unmatched cohorts and **(B)** matched cohorts. *The hazard ratio was estimated from the survival curves.

Some studies managed to balance the baseline characteristics to overcome selection bias by paired design or propensity score matching. Of the nine studies included, three were unmatched studies (20, 21, 24), another six were matched studies (22, 23, 25–28), with four of which reporting the comparing outcomes before matching (22, 23, 25, 26). Finally, seven unmatched cohorts and six matched cohorts were included in the pooled analysis. The characteristics of BCLM patients in the unmatched and matched cohorts were summarized in Tables 2, 3, respectively. Surgical treatment combined with systemic treatment was associated with better OS than systemic treatment alone in the pooled analysis of unmatched cohorts (HR = 0.58, 95%CI: 0.36 to 0.92, I2 = 88%; Figure 3A). The result of the LR subset was consistent (HR = 0.53, 95%CI: 0.31 to 0.89, I2 = 89%; Figure 3A). We also came to the consistent results in the pooled analysis of matched cohorts with a pooled HR of 0.46 (95%CI: 0.36 to 0.59, I2 = 45%; Figure 3B), and in the subgroup analysis of the LR subset with a pooled HR of 0.42 (95%CI: 0.32 to 0.55, I2 = 50%; Figure 3B).

### Publication Bias

**Figure 4:**
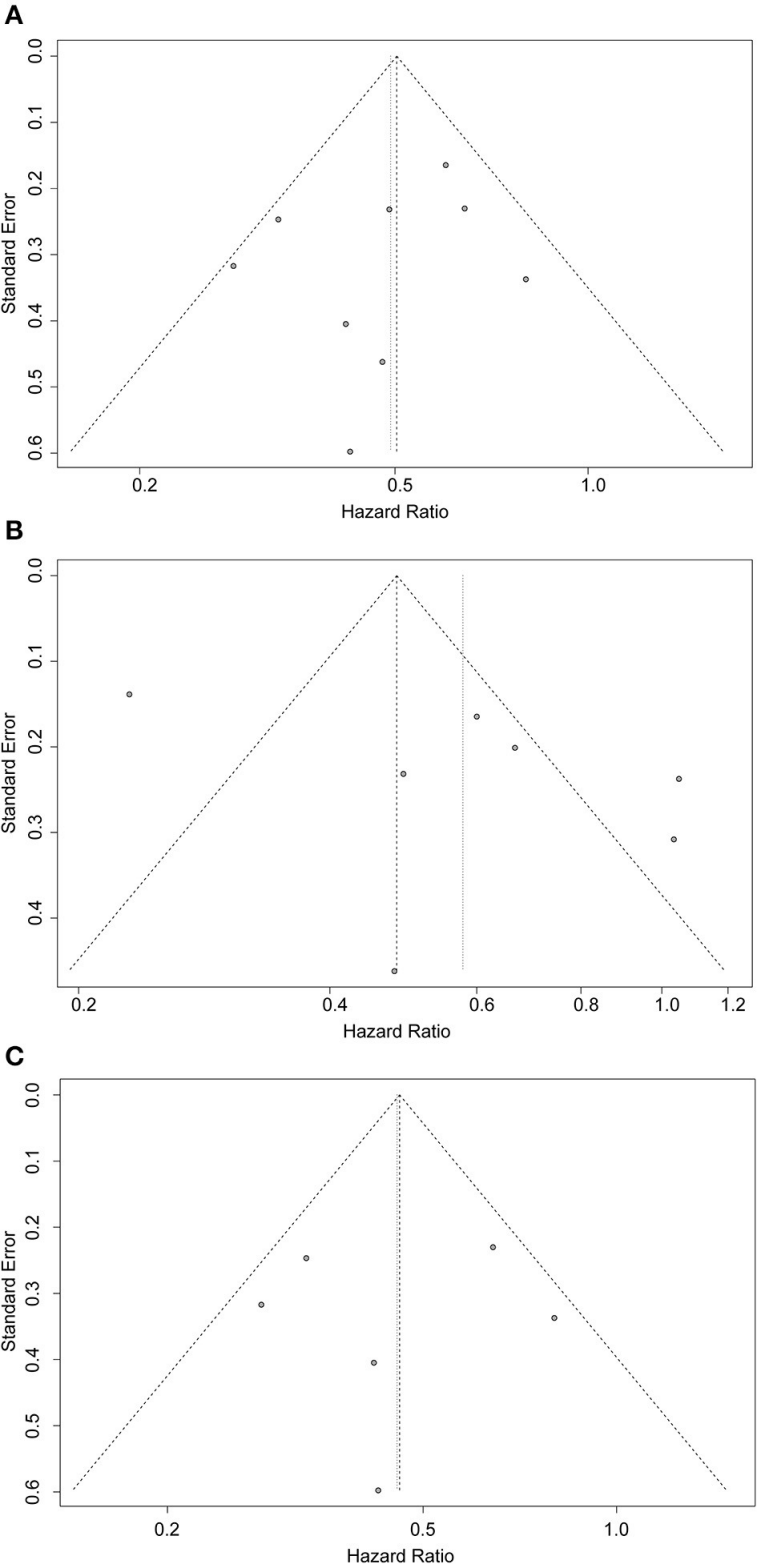
Funnel plots for evaluating publication bias of **(A)** all included studies, **(B)** unmatched cohorts, and **(C)** matched cohorts.

As shown in Figure 4, the possible publication bias was assessed by the funnel plot, Egger's test, and Begg's test. The funnel plot for all the included studies was symmetrically distributed, and none of the studies was outside the 95%CI (Figure 4A). No significant publication bias was detected from statistical tests based on OS (Begg's test P = 0.4042; Egger's test P = 0.4305). The funnel plots for the unmatched (Figure 4B) and matched (Figure 4C) cohorts were also symmetrical.

## Discussion

BCLM is common in advanced breast cancer, and generally carries a poor prognosis. Systemic therapy has become the standard treatment for patients with disseminated disease (3). However, noting the curative potential of surgical treatment for CRLM, some clinicians have advocated surgical treatment for a select group of patients with isolated BCLM (8–10). There is growing evidence that surgical treatment may contribute not only to assessing histological information and the subtype of metastatic tumors, while also providing potential de-escalation of toxic systemic therapies and prolonging the time before more aggressive systemic therapy needs to be initiated (29). Nevertheless, experts have not reached a definitive consensus on this topic. The ESO-ESMO International Consensus Guidelines for Advanced Breast Cancer note that surgical treatment for BCLM is considered in select patients (3), but the National Comprehensive Cancer Network Guidelines do not recommend surgical treatment as an option (30). This is due to the lack of randomized data supporting the prognostic effect of surgical treatment and the inconsistent results of published retrospective studies. The reason for these disparate results is undoubtedly multifactorial. Most studies on this topic were retrospective, derived from small case series, and were highly susceptible to the effects of selection bias and confounding. Thus, an updated systematic review and meta-analysis was necessary to ascertain the prognostic value of surgical treatment for isolated BCLM.

There are many approaches of local treatment, however, sole focus was put on LR and RFA in our study, this choice was made because of the implications of previous studies. Local treatment is now an established part of the treatment of CRLM because it is associated with extended survival and therapeutic potential (31). Among the methods of local treatment, specifically LR and RFA have the most exact efficacy, because they have the most reported prognostic benefits for patients with CRLM. This fact, coupled with the improvement in the safety of surgical treatment in high-level medical centers, has led to an increase in the number of LR and RFA for non-colorectal LM, specifically isolated BCLM (26, 27, 32–36). In a recent systematic review of surgical treatment for isolated BCLM consisting of 43 studies and 1,686 patients, the median OS was 36 months (12–58 months) and the median 1-, 3-, and 5-year OS were 90, 56, and 37%, respectively (10). Therefore, some have advocated that LR and RFA should follow a clinical application similar to CRLM for selected patients with isolated BCLM (37, 38). Our study hereby focused on the prognostic value of LR and RFA for patients with isolated BCLM, in order to provide a basis for the promotion of surgical treatment to isolated BCLM.

Our study confirmed that systemic treatment combined with surgical treatment for isolated BCLM was associated with a better prognosis than systemic treatment alone. We conducted subgroup analyses by categorizing the approaches of surgical treatment to improve the reliability of the conclusions, and the results of the LR subset were consistent with those in the whole cohorts. Besides, intriguing features of patients who received surgical treatment could be drawn out from Tables 2, 3, they were more likely to be young at diagnosis, have solitary distribution of liver lesions, have longer intervals from breast cancer diagnosis to BCLM, and respond better to systemic therapy. Previous studies also found some similar features among surgically treated patients with BCLM, e.g. unilobar distributed lesions, normal liver function, and good performance statuses (8–10, 29). These unique characteristics might represent the selective criteria of surgical treatment for patients with isolated BCLM in clinical practice (22, 28). They represented an early or intermediate state in the progression of BCLM when potential cure may be easily achieved by multidisciplinary treatment. When comparing systemic therapy alone vs. systemic therapy combined with surgical therapy, one must keep in mind that the superior outcomes in the latter might stem partially from the potential selection bias and not necessarily from the surgical treatment *per se*. In some cases, some patients were selected for surgical treatment because they responded well to systemic therapy, which was aimed either to convert initially unresectable disease or to test tumor biology, as was the case in CRLM. In turn, these patients exhibited better prognoses not because of the surgical treatment but because of their more indolent biology. This may explain the disparate results of some studies in this meta-analysis: the Sadot et al.'s study (26) which found comparable 5-year OS in surgically vs. systemically treated patients with BCLM, and the studies of Feng et al. and Ruiz et al. (22, 25) which found that surgically resected patients had improved OS compared to those treated with systemic therapy. Indeed, in the latter two studies, the surgical groups had up to 100% proportions of patients who had stable disease control after adequate systemic therapy. Therefore, the highly selected indications for surgical treatment for isolated BCLM will inevitably lead to selection bias. Due to the fact that only two studies (23, 28) strictly matched the response rate to systemic treatment between the groups, a subgroup analysis by response to systemic treatment would not be feasible. To overcome the effects of selection bias, some studies have managed to balance the baseline characteristics of surgical and medical cohorts with some methods, including paired design and propensity score matching (22, 23, 25–28). In our study, we combined the results of the matched and unmatched cohorts separately to verify our conclusions. In view of the limited number of related studies and their retrospective design, to our knowledge, this was the approach to minimize the baseline differences between the groups as far as practicable. The results of pooled analysis of matched cohorts still strongly supported the prognostic benefit of surgical treatment, which was more convincing and credible because of the minimization of selection bias, considering that the baseline characteristics of the two groups have been balanced.

Our study has several notable strengths: (I) This is the first pooled meta-analysis to confirm the survival advantage of surgical treatment for patients with isolated BCLM. Previous articles on this topic were all systematic review, and no pooled analysis has been done. This was because most of the related studies published before 2020 were single-arm case series, with no comparing results reported. It was the five related original articles with comparing results published after 2020 that made pooled analysis possible (20–24). Therefore, our conclusion is the highest level of evidence to date on the prognostic benefits of surgical treatment for isolated BCLM, since there are no relevant randomized controlled trials published until now. (II) Many previous studies claiming the survival advantage of surgical treatment for metastatic cancer were based on a comparison of the 1/3/5-year survival rates between groups (39–42). Its major disadvantage lied in the fact that it only focused on the difference in survival rate at some specific time points, but couldn't represent the long-term prognosis. This study overcame this defect by incorporating the HRs into the meta-analysis. (III) We further performed the pooled analysis of the matched and unmatched cohorts respectively to eliminate confounding variables associated with selection bias.

Our study also has some limitations: (I) Due to the limited quantity and different emphases of included studies, it was not feasible to conduct other subgroup analysis which was of more clinical significance, such as based on the response to chemotherapy. Similarly, it was also not feasible to choose other survival outcomes as the primary endpoints, such as progression-free survival. (II) We estimated some HRs from the survival curves in some studies which did not report HRs (22, 23, 26, 27), and this method of estimating HRs might lead to a more conservative *P* value and a higher possibility of committing type-2 errors. (III) Matching variables differed in studies, some studies did not include the key variables we identified (28), some did not report whether these variables were balanced after matching (26, 28), which might be a potential source of bias.

## Conclusion

Compared with systemic treatment alone, surgical treatment combined with systemic treatment was proven to be associated with superior survival outcomes, which should be considered in selected patients with isolated BCLM. Further randomized controlled trials are needed to confirm our conclusions.

## Data Availability Statement

The original contributions presented in the study are included in the article/Supplementary Material, further inquiries can be directed to the corresponding author/s.

## Ethics Statement

All the procedures followed were in accordance with the Helsinki Declaration of the World Medical Association (as revised in 2013). No ethical clearance or informed consent was necessary, since there was no intervention or interaction with humans.

## Author Contributions

All authors: Conception and design and manuscript writing. M-SS and H-JL: Collection and assembly of data and Data analysis and interpretation. All authors contributed to the article and approved the submitted version.

## Funding

This study was supported by National Key R&D Program of China (2016YFC0901302) and Interdisciplinary Clinical Research Project of Peking University First Hospital (2019CR38).

## Conflict of Interest

The authors declare that the research was conducted in the absence of any commercial or financial relationships that could be construed as a potential conflict of interest.

## Publisher's Note

All claims expressed in this article are solely those of the authors and do not necessarily represent those of their affiliated organizations, or those of the publisher, the editors and the reviewers. Any product that may be evaluated in this article, or claim that may be made by its manufacturer, is not guaranteed or endorsed by the publisher.
