# Supplementary material for: Survival Benefit of Surgical Treatment Added Into Systemic Treatment for Isolated Breast Cancer Liver Metastases: A Systematic Review and Meta-Analysis
Source: Front Surg. 2021 Oct 25;8:751906. doi: 10.3389/fsurg.2021.751906 (PMC8573018; doi:10.3389/fsurg.2021.751906)
Supplement: Supplementary file 1 [file Data_Sheet_1.docx]

Supplementary Material

# Full Electronic Search Strategies

## PubMed Search Strategy

((((((((((((("Hepatectomy"[Mesh]) OR ("Metastasectomy"[Mesh])) OR ("Radiofrequency Ablation"[Mesh])) OR (Hepatectomy[Title/Abstract])) OR (metastasectomy[Title/Abstract])) OR (resection[Title/Abstract])) OR (ablation[Title/Abstract])) OR (ablative[Title/Abstract])) OR (microwave[Title/Abstract])) OR (surgery[Title/Abstract])) OR (surgical[Title/Abstract])) AND (((("Neoplasm Metastasis"[Mesh]) OR (metastasis[Title/Abstract])) OR (metastases[Title/Abstract])) OR (metastatic[Title/Abstract]))) AND ((("Liver"[Mesh]) OR (liver[Title/Abstract])) OR (hepatic[Title/Abstract]))) AND (("Breast Neoplasms"[Mesh]) OR (breast[Title/Abstract]))

## Embase Search Strategy

#1: 'breast tumor'/exp

#2: breast: ab, ti

#3: #1 OR #2

#4: 'liver'/exp

#5: liver: ab, ti

#6: hepatic: ab, ti

#7: 'liver disease'/exp

#8: #4 OR #5 OR #6 OR #7

#9: 'metastasis'/exp

#10: metastasis: ab, ti

#11: metastases: ab, ti

#12: metastatic: ab, ti

#13: #9 OR #10 OR #11 OR #12

#14: 'liver resection'/exp

#15: 'metastasis resection'/exp

#16: 'radiofrequency ablation'/exp

#17: hepatectomy: ab, ti

#18: metastasectomy: ab, ti

#19: resection: ab, ti

#20: ablation: ab, ti

#21: ablative: ab, ti

#22: microwave: ab, ti

#23: surgery: ab, ti

#24: surgical: ab, ti

#25: #14 OR #15 OR #16 OR #17 OR #18 OR #19 OR #20 OR #21 OR #22 OR #23 OR #24

#26: #3 AND #8 AND #13 AND #25

## Cochrane Library Search Strategy

#1: MeSH descriptor: [Breast Neoplasms] explode all trees

#2: (breast): ti, ab, kw

#3: MeSH descriptor: [Liver] explode all trees

#4: (liver): ti, ab, kw

#5: (hepatic): ti, ab, kw

#6: MeSH descriptor: [Neoplasm Metastasis] explode all trees

#7: (metastasis): ti, ab, kw

#8: (metastases): ti, ab, kw

#9: (metastatic): ti, ab, kw

#10: MeSH descriptor: [Hepatectomy] explode all trees

#11: MeSH descriptor: [Metastasectomy] explode all trees

#12: MeSH descriptor: [Radiofrequency Ablation] explode all trees

#13: (hepatectomy): ti, ab, kw

#14: (metastasectomy): ti, ab, kw

#15: (resection): ti, ab, kw

#16: (ablation): ti, ab, kw

#17: (ablative): ti, ab, kw

#18: (microwave): ti, ab, kw

#19: (surgery): ti, ab, kw

#20: (surgical): ti, ab, kw

#21: #1 OR #2

#22: #3 OR #4 OR #5

#23: #6 OR #7 OR #8 OR #9

#24: #10 OR #11 OR #12 OR #13 OR #14 OR #15 OR #16 OR #17 OR #18 OR #19 OR #20

#25: #21 AND #22 AND #23 AND #24

# Supplementary Table 1. Results of Other Survival Outcomes in the Included Studies^#^.

| Study | Definition of other survival outcomes | Results of other survival outcomes |
| --- | --- | --- |
| Bilani 2020 | -- | -- |
| Chun 2020 | RFS was calculated from date of LR to disease relapse for the surgical group.  PFS was calculated from date of LR to disease relapse or death for the surgical group. | Before matching, the median RFS was 14 (range, 1-89) months in the surgical group.  Before matching, the median PFS was 60 (95% CI, 9-111) months, 17 (95% CI, 12-22) months, 16 (95% CI, 0-40) months, and 5 (95% CI, 3-7) months in the surgical group of HER2-enriched, Luminal A, Luminal B, and triple-negative breast cancer, respectively. |
| Feng 2020 | Intrahepatic RFS was calculated from date of LR to recurrence in liver for the surgical group.  Intrahepatic PFS was calculated from date of medical treatment to progression in liver for the systemic group. | Before matching, the median RFS was 32.0 (95% CI, 17.3-46.7) months in the surgical group. The 1-, 3- and 5-year RFS rates were 90.4%, 66.5% and 41.2%, respectively.  After matching, the median RFS was 32.0 (95% CI, 10.2-53.8) months in the surgical group. The 1-, 3- and 5-year RFS rates were 73.2%, 41.1% and 41.1%, respectively.  Before matching, the median PFS was 7.5 (95% CI, 6.6-8.4) months in the systemic group. The 1-, 3- and 5-year PFS were 32.1%, 7.4% and 4.3%, respectively.  After matching, the median PFS was 6.0 (95% CI, 4.5-7.5) months in the systemic group. The 1-, 3- and 5-year PFS rates were 28.8%, 9.5% and 3.8%, respectively. |
| Mariani 2013 | -- | -- |
| Millen 2020 | -- | -- |
| Ruiz 2018 | -- | -- |
| Sadot 2016 | RFS was calculated from date of LM diagnosis to disease recurrence for the surgical group. | Before matching, the median RFS was 28.5 (95% CI, 19-38) months in the surgical group. |
| Sunden 2020 | -- | -- |
| Tasci 2013 | DFS was calculated from date of LM diagnosis for the surgical group, the end points were not clearly defined. | The median DFS was 9 months in the surgical group. |

^#^Overall survival was the primary survival outcome of all the included studies and was summarized in Table 2-3. Therefore, we summarized the results of all the other survival outcomes except overall survival in this supplementary table.

Abbreviations: RFS, recurrence-free survival; PFS, progression-free survival; DFS, disease-free survival; LR, liver resection; LM, liver metastases; HER2, human epidermal growth factor receptor 2.
